# Factors influencing early postnatal care utilisation among women: Evidence from the 2014 Ghana Demographic and Health Survey

**DOI:** 10.1371/journal.pone.0249480

**Published:** 2021-04-02

**Authors:** Francis Appiah, Tarif Salihu, Justice Ofosu Darko Fenteng, Andrews Ohene Darteh, Esther Twewa Djan, Matthew Takyi, Patience Ansomah Ayerakwah, Edward Kwabena Ameyaw

**Affiliations:** 1 Department of Population and Health, University of Cape Coast, Cape Coast, Ghana; 2 Berekum College of Education, Berekum, Bono Region, Ghana; 3 Department of Optometry, University of Cape Coast, Cape Coast, Ghana; 4 School of Public Health, Faculty of Health, University of Technology Sydney, Sydney, Australia; University of Mississippi Medical Center, UNITED STATES

## Abstract

**Introduction:**

Early postnatal care (EPNC) utilisation is crucial for averting maternal deaths as recommended by the World Health Organisation. About 30% of women do not obtain EPNC in Ghana and no national level study have investigated the determinants of EPNC. Therefore, this study aimed at assessing factors associated with EPNC uptake among women aged 15–49 in Ghana.

**Materials and methods:**

The study utilised data from the women’s file of the 2014 Ghana Demographic and Health Survey (GDHS) and sampled 1,678 women aged 15–49 who had complete data on EPNC. Descriptive computation of EPNC was done. Since EPNC (which is the main outcome variable for the study) was dichotomous, the binary logistic regression was used to determine factors influencing utilisation of EPNC at 95% two-tailed confidence interval. The results were presented as adjusted odds ratio (AOR). Stata version 14.0 was used for all the analyses.

**Results:**

Descriptively, the results indicated that 31% of women aged 15–49 sought EPNC. At the inferential level, women aged 40–44 were more likely to seek EPNC compared to those aged 15–19 [AOR = 3.66, CI = 1.25–10.67]. Islam women had higher odds of EPNC as compared with Christians [AOR = 1.70, CI = 1.23–2.35]. Comparatively, women of Mande ethnic group had higher propensity to seek EPNC than the Akan [AOR = 3.22, CI = 1.20–8.69]. Residents of the Greater Accra region were over 11 times probable to utilise EPNC compared with the residents of Western region.

**Conclusion:**

The key determinants of EPNC were age, religion, ethnicity, marital status and region. Therefore, the Health Promotion and Education Unit and Reproductive and Child Health Department of the Ghana Health Service need to scale up EPNC sensitisation programmes and should target women aged 15–19, Christians and other category of women with less likelihood of EPNC in order to offset the disparities.

## Introduction

Worldwide, 61% of the 585,000 average maternal deaths witnessed every year transpire within the postnatal stage [1] and sub Saharan Africa (SSA) bears the highest share of these deaths. In 2017, SSA recorded about 66% of the global maternal deaths (533 maternal deaths per 100,000 live births) [2, 3]. The situation is almost same in the Ghanaian context as maternal deaths remains high, averaging 310 maternal deaths per 100,000 live births in 2017 [4]. More so, about 50% of maternal deaths in Ghana emerge within the first 24 hours after birth [5]. This can be averted if mothers obtain early and timely postnatal care offered by skilled health personnel [6–8]. Thus postnatal complications such as birth asphyxia, trauma and sepsis are manageable through comprehensive early postnatal care (EPNC) which guarantees timely diagnoses for danger signs in infant’s breathing, temperature and breastfeeding [9].

EPNC is the first postpartum check women receive from healthcare providers within 24 hours after birth [9, 10] and is crucial for averting maternal deaths [8]. Consequently, the World Health Organization [WHO] recommends that mothers should have EPNC within 24 hours after childbirth and three additional postnatal care (PNC) visits (which should fall within 48–72 hours, 7–14 days, and before 6 weeks after delivery) [9, 10]. In line with this recommendation, Ghana has introduced several maternal health policies partly to strengthen maternal healthcare. Typically, the National Health Insurance Scheme (NHIS) and the Free Maternal Health Policy (FMHP) introduced in 2003 and 2008 respectively aimed at reducing maternal and child mortality by eliminating the financial barrier to maternal and child healthcare [11–13]. Additionally, women who are subscribed to the NHIS enjoy free antenatal care, delivery service and postnatal care. The free postnatal services comprise free consultation and medicines for two postnatal visits [14, 15].

Regardless of the policies on EPNC and maternal healthcare, some women do not utilise EPNC services in Ghana [16]. About 30% women are unable to seek EPNC in Ghana [16]. On the same token, the Ghanaian literature have focused on predictors of postnatal care attendance in general but not on determinants of EPNC. For instance, the Ghana Statistical Service (GSS), Ghana Health Service (GHS) and ICF reported that urban residents are more inclined to postnatal check-up than rural residents [4]. Sakeah et al. [17] also observed that few mothers in rural areas of West Mamprusi district in Ghana receive the three recommended PNC visits. Utilization of maternal health services (such as PNC) is influenced by age of mother, type of birth, education of mother, ethnicity, economic status, geographic location, residence, and religious affiliation [18]. This has been affirmed by Appiah et al. [19] who revealed that women in Savanna zone of Ghana, the Guan ethnic group, working class and those who consider distance as unproblematic have higher odds of postnatal care compared to their counterparts.

Ndugga et al. [20] assessed determinants to EPNC and found that delivery at a health facility was the most important determinant of EPNC attendance. However, their study was conducted in Uganda, hence their findings might not be wholly applicable in Ghana. As a results, there is a knowledge gap on determinants of EPNC in Ghana (i.e. PNC within 24 hours). Therefore, this study seeks to determine the factors associated with EPNC among women aged 15–49 in Ghana. The study is relevant for health policy direction. The health promotion and education unit as well as the reproductive and child health department of the Ghana Health Service will find this study useful in their policy decisions regarding maternal and newborn healthcare delivery. The study seeks to address this question: What influence EPNC among women aged 15–49 in Ghana?

**Fig 1:**
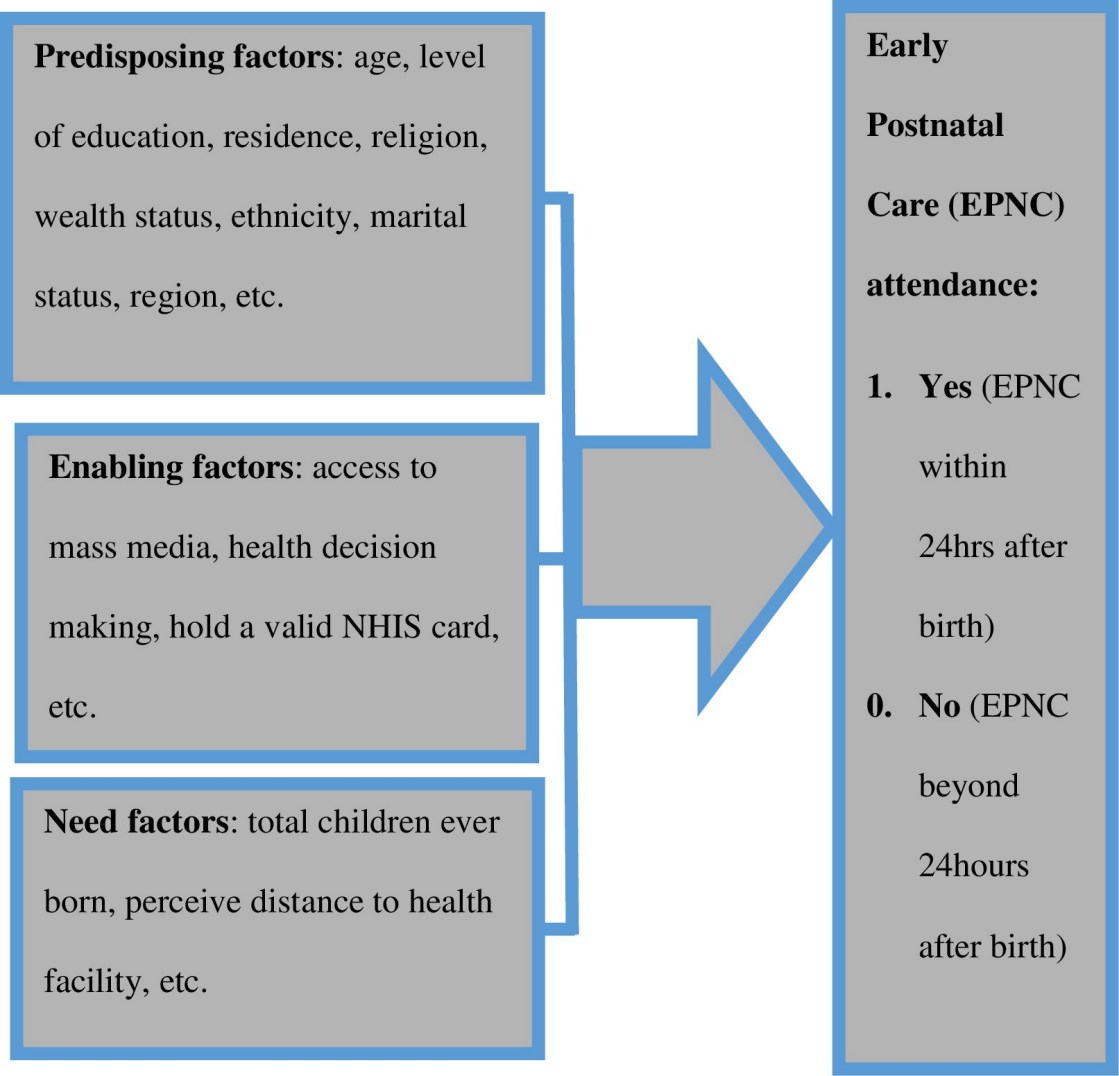
A modified version of Andersen’s healthcare utilisation model. Source (Andersen, 1995; Ndugga et al., 2020).

Anderson’s healthcare utilisation model shall be used as a guide for this study in order to address this question appropriately [20–23]. The model purports that healthcare utilisation is determined by predisposing, enabling and need factors [23, 24]. According to the model, the predisposing factors are demographics and social structures whereas the enabling factors are those that facilitate individuals to use services. Such enabling factors include access to insurance, income, access to free services as well as availability and access to the service [25]. The need factors also stress the factors that motivate service use including physical conditions, illness or disease conditions [25]. In this study, it is anticipated that EPNC uptake shall be determined by predisposing factors (including age, level of education, residence, religion, wealth status, ethnicity, marital status, region); enabling factors (such as access to mass media, health decision making, hold a valid NHIS card); and need factors (such as total children ever born and perceive distance to health facility) (Fig 1). The model provided a robust analytical framework for explaining drivers to EPNC uptake [20].

## Materials and methods

### Data source

The study utilised women’s file of the 2014 Ghana Demographic and Health Survey (GDHS). The 2014 GDHS, which is the current and sixth edition of the surveys captures information on maternal and child health including place of delivery, ANC utilisation, feeding practices of children under-five, women’s reproductive performance, family planning and other information relevant for policy formulation. The Inner-City Fund (ICF) International offered technical support through the DHS Program, however, the survey was jointly implemented by the Ghana Statistical Service (GSS), the Ghana Health Service (GHS), and the National Public Health Reference Laboratory (NPHRL) of the GHS. The data were gathered with the Demographic and Health Survey (DHS) standardised questionnaire which is developed by the Measure DHS programme [16].

The survey used an updated sampling frame that dwelled on the existing sampling frame developed by the GSS for the 2010 Population and Housing Census (PHC) activity [16]. The survey followed a stratified sampling procedure and selected 9,658 eligible women from the identified households. In all, 9,396 women were interviewed, with a response rate of 97.3%. This study was restricted to 1,678 women aged 15–49 who had complete data about the variables of study. Since the sampling procedure employed in the survey was probabilistic, the sample was not self-weighting at the national level, however, weighting factor was added to the data file so that the results will be proportional at the national level. Details of sampling procedure and fieldwork issues are documented in the 2014 GDHS report [16].

### Derivation of outcome variable

The World Health Organization (WHO) recommends that mothers should receive their first PNC within the first 24 hours after delivery [9, 10]. As a results, women aged 15–49 who had a birth in the 5 years before the 2014 GDHS were asked about the time postnatal check took place after delivery. Therefore, the outcome variable for this study was ‘‘Early Postnatal Care”, defined as having received a postnatal check within 24 hours of delivery. ‘No’ responses were coded as ‘0’ to indicate those who received PNC check after 24 hours and ‘Yes’ were coded as ‘1’ to denote those who had PNC within 24 hours after delivery. For certainty in responses, women who affirmed ‘don’t know’ were excluded from the analysis. Women who delivered through caesarean section (CS) are likely to spend several days at health facilities to ensure smooth recovery and management of further complications that might arise; hence, they are likely to have early and consistent PNC checks and were excluded in conformity with a previous study [20].

### Derivation of independent variables

Fourteen independent variables of theoretical importance to EPNC and maternal healthcare utilisation were selected for the study [20, 26]. These are age, level of education, residence, religion, wealth status, ethnicity, marital status, region, total children ever born, partner’s level of education, access to mass media, health decision making, hold a valid NHIS card, getting medical help for self: distance to health facility. In order to generate reader friendly results, level of education was recoded as ‘no education’, ‘primary’ and ‘secondary or higher’. Occupation was recoded as ‘not working’ and ‘working’ whereas religion of affiliation also recoded as ‘Christian’, ‘Islam’, ‘Traditionalist’ and ‘No religion’. Considering recent fertility rate of Ghana (which is 4.2 children per woman) [16], total children ever born was recoded as ‘one birth’, ‘two births’, ‘three births’ and ‘four or more births’. Moreover, partner’s education was recoded as ‘no education’, ‘primary’ and ‘secondary or above’ whereas health decision-making capacity was also recoded as ‘alone’ and ‘not alone’. Finally, access to mass media was obtained from three prime variables: frequency of reading newspaper/magazine; frequency of listening to radio; and frequency of watching television. Each of these variables had three responses: ‘not at all’, ‘less than once a week’ and ‘at least once a week’. We combined ‘less than once a week’ and ‘at least once a week’ as having access to mass media whilst ‘not at all’ was considered as not having access to mass media.

### Statistical analysis

**Fig 2:**
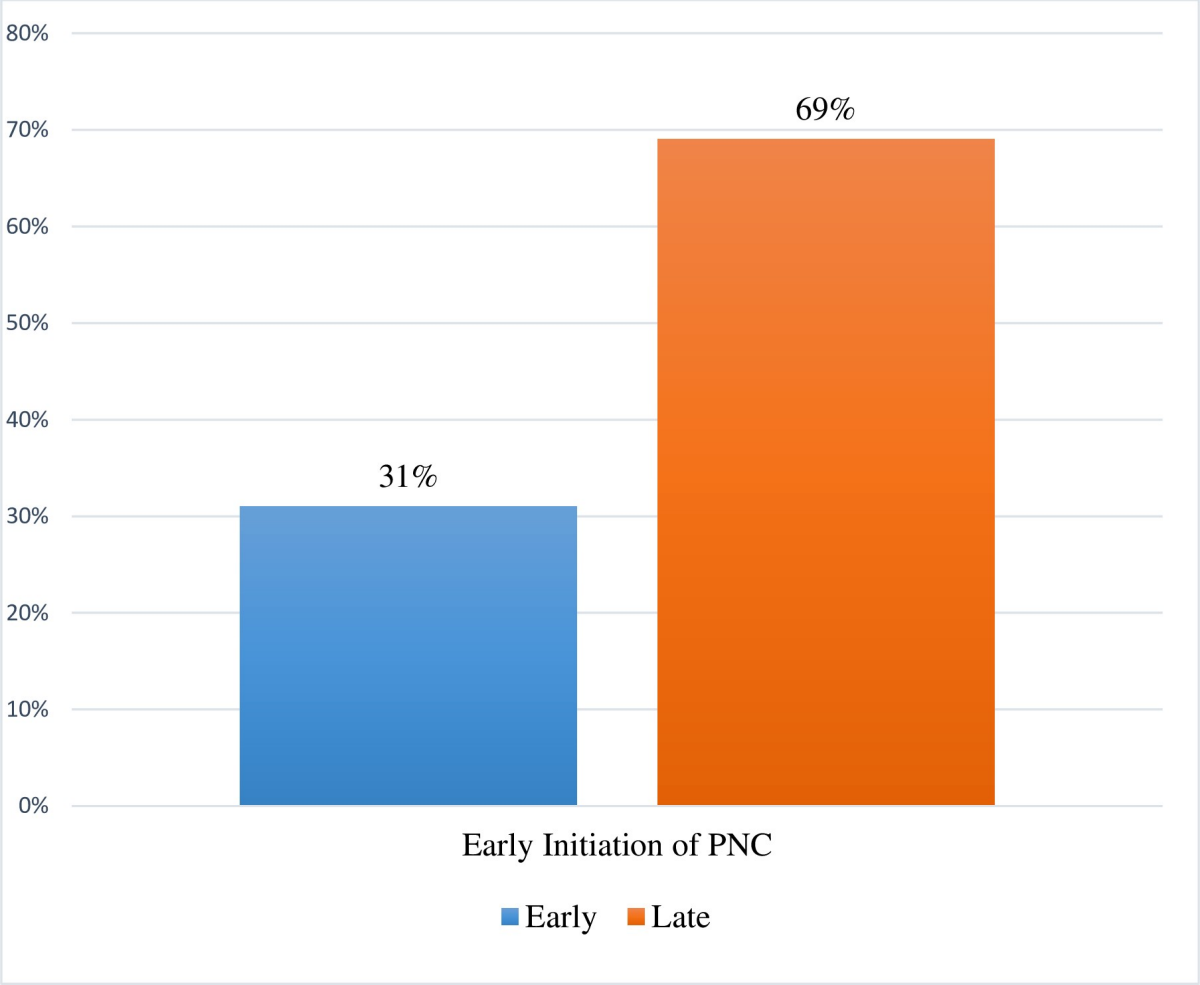
Distribution of early initiation of PNC in Ghana. Computed from 2014 GDHS.

The following analytical procedures were followed. Firstly, we calculated the proportion of women aged 15–49 in Ghana who had EPNC or otherwise and the results were presented using bar graph (Fig 2). After that, a bivariate computation of EPNC and the selected explanatory variables with their chi square test of independence reported and the results were presented in proportions and percentages (Table 1). Further, we applied variance inflation factor (VIF) to test for multi-collinearity between our explanatory variables and the results indicated that our independent variables were not highly correlated (Mean VIF = 1.56, Maximum VIF = 3.05, Minimum VIF = 1.02) (S1 Appendix). Thereafter, we employed the binary logistic regression to determine factors influencing utilisation of early PNC. The binary logistic regression was selected since our outcome variable was dichotomous in nature. We set the significance at 95% alpha threshold at two-tailed confidence interval and the results of the model presented in an adjusted odds ratio (AOR). Our results were interpreted to be less likelihood to early PNC utilisation when the AOR was less than one and increased likelihood to early PNC if the AOR was above one. We applied the weighting factor (v005/100000) inherent in the dataset to offset the complex survey sampling errors. Additionally, the ‘linktest’ command was applied to assess the fitness of our model (S2 Appendix) and Stata version 14.0 was used for all our analyses.

**Table 1:** Socio-demographic characteristics and EPNC (N = 1,678).

Socio-demographic	Weighted (N)	Weighted (%)	Early initiation of PNC		X^2^(p-value)
			Late(%)	Early(%)	
**Age**					8.4299(0.208)
15–19	38	3	82	18	
20–24	242	14	67	33	
25–29	451	27	68	32	
30–34	416	24	64	36	
35–39	341	20	70	30	
40–44	145	9	64	36	
45–49	45	3	73	27	
**Education**					1.8237(0.402)
No education	539	32	66	34	
Primary	272	16	66	34	
Secondary+	867	52	69	31	
**Residence**					1.5928(0.207)
Urban	750	45	66	34	
Rural	928	55	69	31	
**Religion**					33.9062(0.000)
Christian	1167	70	69	31	
Islam	416	25	59	41	
Traditionalist	46	2	78	22	
No religion	49	3	91	9	
**Wealth status**					6.3445(0.173)
Poorest	451	27	66	34	
Poorer	311	18	72	28	
Middle	265	16	67	33	
Richer	303	18	69	31	
Richest	348	21	63	37	
**Ethnicity**					105.6262(0.000)
Akan	619	36	75	25	
Ga/Dangme	74	5	52	48	
Ewe	232	13	72	28	
Guan	44	3	80	20	
Mole-Dagbani	424	26	57	43	
Grusi	74	4	61	39	
Gurma	156	9	88	12	
Mande	21	1	31	69	
Other	34	3	69	31	
**Marital status**					20.7887(0.000)
Married	1333	79	65	35	
Cohabiting	345	21	79	21	
**Region**					209.4637(0.000)
Western	162	10	88	12	
Central	144	8	60	40	
Greater Accra	277	16	44	56	
Volta	171	11	79	21	
Eastern	60	4	76	24	
Ashanti	228	14	80	20	
Brong Ahafo	128	8	80	20	
Northern	315	19	81	19	
Upper East	113	6	52	48	
Upper West	80	4	43	57	
**Occupation**					0.5360(0.464)
Not working	265	15	65	35	
Working	1413	85	68	32	
**Total children ever born**					0.2967(0.961)
One birth	283	16	68	32	
Two births	370	23	68	32	
Three births	331	19	66	34	
Four or more births	694	42	67	33	
**Partners’ education**					4.3952(0.111)
No education	422	25	67	33	
Primary	182	11	61	39	
Secondary+	1074	64	69	31	
**Access to Mass Media**					0.0860(0.769
No	565	34	67	33	
Yes	1113	66	67	33	
**Health decision making**					0.4447(0.505)
Alone	386	23	69	31	
Not alone	1292	77	67	33	
**Hold a valid NHIS card**					0.0271(0.869)
No	247	15	68	32	
Yes	1431	85	67	33	
**Getting medical help for self: distance to health facility**					4.6636(0.031)
Big problem	426	26	71	29	
Not a big problem	1252	74	66	34	

Deduced from 2014 GDHS

### Ethical considerations

Since the authors did not participate in the actual data gathering, no ethical clearance was sought for this study. However, permission to use the data set from MEASURE DHS was sought and after they have assessed our intent for using the dataset, permission to use the data was granted. Meanwhile, Measure DHS reported that ethical clearance was obtained from the Institutional Review Board of ICF International and Ethical Review Committee of Ghana Health Service [16]. The data set underpinning this study is publicly available at www.measuredhs.org.

## Results

### Descriptive results for the study

From Fig 2, we realised that 31% of women aged 15–49 sought EPNC. From Table 1, not more than 40 percent attended PNC early, especially among those aged 30–34 (36%) and 40–44 (36%). The least to have utilise PNC early were those with secondary or higher education (31%). Whereas 34% of women in urban areas initiated PNC early, 41% of the Muslims initiated PNC early.

It was found that just a little over one-third of those aged 30–34 (36%) and 40–44 (36%) had EPNC. Thirty-four percent among those with no education and primary education utilised EPNC and similar was recorded among urban residents (34%). Also, 41% of the Islam obtained EPNC whereas 37% of the richest utilised EPNC. The results indicated that over two-thirds of the Mande ethnic group (69%) attended PNC early whilst a little above one-third of the married (35%) had EPNC (Table 1).

Over half of the women in the Upper West region (57%) obtained EPNC whilst 35% of those not working had EPNC. Thirty-four percent of women with two births had EPNC whereas 39% of those whose partners completed primary education attended PNC early. Thirty-three percent of women who had access to mass media sought EPNC. Similarly, 33% of those who had a valid NHIS attended PNC early. Also, a little above one-third of those who perceived distance to health facility as not a big problem (34%) utilised EPNC. The chi square test for independence indicated that only ‘getting medical help for self: distance to health facility’, ‘region’, ‘marital status’, ‘ethnicity’, and ‘religion’ had statistical association with the outcome variable (Table 1).

### Inferential results for the study

Table 2 presents the inferential results for the study. Women aged 40–44 were 3.66 times higher to initiate PNC early as compared to those aged 15–19 [AOR = 3.66, CI = 1.25–10.66]. Compared with Christians, Islam women had high propensity to initiate PNC early [AOR = 1.70, CI = 1.23–2.35], just as among the Mande ethnic group compared with the Akan ethnic group [AOR = 3.22, CI = 1.19–8.69]. Those cohabiting had less odds of utilising PNC early compared to the married [AOR = 0.68, CI = 0.47–0.97]. Moreover, residents of the Greater Accra region were over 11 times higher to initiate PNC early compared with the residents of Western region [AOR = 11.13, CI = 5.48–22.60]. Finally, from the model specification test, it was evident that our model was well-specified (S2 Appendix).

**Table 2:** Binary logistic regression results.

Socio-demographics	AOR	95% CI
**Age**		
15–19	Ref	1,1
20–24	3.42*	[1.32–8.89]
25–29	3.13*	[1.20–8.16]
30–34	3.45*	[1.27–9.37]
35–39	2.87*	[1.03–7.96]
40–44	3.66*	[1.25–10.66]
45–49	2.20	[0.66–7.31]
**Education**		
No education	Ref	1,1
Primary	0.99	[0.70–1.41]
Secondary+	0.91	[0.64–1.30]
**Residence**		
Urban	Ref	1,1
Rural	1.11	[0.80–1.53]
**Religion**		
Christian	Ref	1,1
Islam	1.70***	[1.23–2.35]
Traditionalist	0.61	[0.30–1.26]
No religion	0.26**	[0.10–0.69]
**Wealth status**		
Poorest	Ref	1,1
Poorer	0.89	[0.61–1.30]
Middle	1.24	[0.79–1.94]
Richer	0.86	[0.51–1.43]
Richest	1.10	[0.59–2.04]
**Ethnicity**		
Akan	Ref	1,1
Ga/Dangme	1.39	[0.68–2.84]
Ewe	1.24	[0.68–2.24]
Guan	0.83	[0.37–1.85]
Mole-Dagbani	1.41	[0.86–2.31]
Grusi	0.85	[0.46–1.57]
Gurma	0.77	[0.40–1.49]
Mande	3.22*	[1.20–8.69]
Other	0.67	[0.25–1.81]
**Marital status**		
Married	Ref	1,1
Cohabiting	0.68*	[0.47–0.97]
**Region**		
Western	Ref	1,1
Central	5.45***	[2.83–10.50]
Greater Accra	11.13***	[5.48–22.60]
Volta	2.14	[0.95–4.81]
Eastern	2.72*	[1.15–6.45]
Ashanti	1.77	[0.86–3.66]
Brong Ahafo	1.62	[0.81–3.24]
Northern	1.44	[0.71–2.93]
Upper East	4.86***	[2.45–9.66]
Upper West	8.93***	[4.43–18.01]
**Occupation**		
Not working	Ref	1,1
Working	0.83	[0.60–1.14]
**Total children ever born**		
One birth	Ref	1,1
Two births	1.00	[0.68–1.48]
Three births	1.04	[0.68–1.60]
Four or more births	1.06	[0.67–1.68]
**Partner’s education**		
No education	Ref	1,1
Primary	1.35	[0.91–2.00]
Secondary+	1.19	[0.85–1.68]
**Access to Mass Media**		
No	Ref	1,1
Yes	0.90	[0.68–1.20]
**Health decision making capacity**		
Alone	Ref	1,1
Not alone	0.93	[0.69–1.25]
**Hold a valid NHIS card**		
No	Ref	1,1
Yes	1.31	[0.92–1.87]
**Getting medical help for self: distance to health facility**		
Big problem	Ref	1,1
Not a big problem	1.17	[0.89–1.53]

Sources: GDHS 2014, AOR = Adjusted Odds Ratio, CI = Confidence Interval in square brackets; Ref = Reference Category;

*p<0.05,

**p<0.01,

***p<0.001

## Discussion

EPNC is one of the most effective strategies for improving maternal health outcomes in low and middle-income countries [27]. In line with that, the present study sought to find out the factors that are associated with EPNC utilisation in Ghana. The significant determinants to EPNC were age, religion, ethnicity, marital status and region–all are predisposing factors. Ndugga and colleagues [20] found women’s education level, household wealth status, employment status, antenatal care attendance, place of delivery, whether distance to the health facility is perceived as a problem and access to media as significant determinants to EPNC in Uganda. However, the present study revealed contrasting associated factors and this could be attributable to the differences in study populations. Our results have demonstrated the major determinants to EPNC in Ghana which could at least, guide public health promotion sensitisation programs geared towards EPNC uptake in Ghana. In relation to the theoretical framework used for the study (Fig 1), the subsequent paragraphs discuss the direction of our results that were found significant to EPNC utilisation.

The study revealed that women aged 40–44 were much inclined to EPNC as compared to those aged 15–19. Studies from rural Indonesia, Nigeria and South Sudan noted age as a demographic characteristic which affects maternal healthcare utilization behaviour [28, 29]. A plausible reason to our observation is that, older women might have been exposed to PNC information during their previous ANC visits from their previous births thereby enhancing their knowledge about the need for EPNC. However, Titaley, Dibley and Roberts [28] had earlier reported that older mothers use post-delivery services less frequently than younger mothers in low- and middle-income countries. The results also strengthen the assertion that predisposing factors play a key role in maternal healthcare utilisation [20–23].

Compared with Christian, being an affiliate of Islam increased women’s propensity to initiate PNC early. This affirms that predisposing factors highlighted in the theoretical framework of the study (Fig 1) serve as a determinant to maternal healthcare utilisation [20–23]. In explaining the influence of religion on health-seeking behaviour, Hussen, Tsegaye, Argaw, Andes, Gilliard and del Rio [30] contended that orthodox Christians sometimes rely on spirituality and faith-based practices in seeking healthcare and in coping with illness. Therefore, we reason with Hussen, Tsegaye, Argaw, Andes, Gilliard and del Rio [30] that, the Christians might have relied on their faith, hence prioritising their faith over the recommended PNC by the WHO.

Residents of the Greater Accra region were more likely to initiate PNC early compared with those of Western region. Fotso [31] asserts that improved electricity, transportation, water, and sanitation may enhance a mother’s chance of utilising PNC service. Practically, majority of functional health facilities such as teaching, psychiatric, private, government hospitals among others are located in urban Ghana [32, 33] and in Greater Accra region, which happens to be much urbanised and hosts the national capital. As such, it is not surprising for women hailing from this region to be inclined to early PNC. This finding is plausible because, theoretically, women are able to utilise maternal healthcare when a health facility is close to them as postulated by Andersen’s healthcare utilisation model [20–23].

The current study noted that women of Mande ethnic group were more probable to seek EPNC compared with the Akan. Similarly, the propensity to utilise EPNC differed across ethnic groups in Guatemala [34], China [35] and India [36]. From Ghana, Ganle [37] also indicated that there are widespread maternal healthcare utilisation disparities among the different ethnic groups. Ganle [37] espoused that inability of some women to express themselves when seeking healthcare is due to varied languages spoken by the ethnic groups as well as geographically disadvantaged location of certain ethnic groups in terms of health facility distribution. These factors may explain disparities in maternal healthcare utilisation among the ethnic groups. Although this partly explains our observation, further studies, preferably a qualitative type is needed to understand the phenomenon better.

The present study found that women who were cohabiting were less likely to seek EPNC as compared to the married. The results are in congruent with the theoretical framework that predisposing factors influence the uptake of maternal healthcare utilisation [20–23]. Several reasons could account for this observation. Batalova and Cohen [38] indicated that gender roles are less structured in cohabiting relationships. Moreover, it is known that those cohabiting mostly face misunderstandings than the married as purported by Skinner et al. [39]. As such, it can be adduced that the cohabiting decision-making on EPNC services could induce misunderstanding and less approval from partners. Giving birth out of wedlock is less desired in most localities in Ghana and therefore cohabiting women may least own up to some maternal health services including EPNC as a strategy for averting stigma and disdain.

### Strengths and weaknesses

The novelty of this study stems from the fact that, it is the first of its kind to have investigated factors influencing EPNC utilisation in Ghana. The study made use of cross-sectional survey data, which reflect the views of women aged 15–49 across the entire country and as such, the results and conclusions are based on a nationally representative survey. The rigorous analytical procedures in estimating determinants of EPNC ensured robustness of the results. However, the study has some weaknesses. First, the cross sectional study design does not permit causal inferences to be made. Also, the survey depended on self-reports about timing of initiating PNC from the women’s standpoint. Therefore the possibility of overestimation or underestimation of timing of EPNC may exist. Finally, the study might have been affected by social desirability and recall bias on the part of the surveyed women.

## Conclusions

The main determinants identified to be associated with EPNC include women’s age, religion of affiliation, region of residence, ethnicity and marital status. All these are predisposing factors theoretically. The health promotion and education unit as well as the reproductive and child health department of the Ghana Health Service need to scale up their sensitisation programmes targeted at EPNC among younger women, Christians and other category of women with less likelihood of EPNC in order to bridge the disparities in EPNC utilisation. The realisation that region of residence leads to disparities in propensity of EPNC calls for political commitment towards ensuring equitable distribution of health resources to all regions. Finally, ethnicity was identified to influence EPNC and this warrants further study, preferably using a qualitative design to understand the phenomenon appropriately.

## Supporting information

S1 Appendix Multi-collinearity test results. (DOCX)

S2 Appendix Linktest results. (DOCX)
