# Supplementary material for: Factors influencing early postnatal care utilisation among women: Evidence from the 2014 Ghana Demographic and Health Survey
Source: PLoS One. 2021 Apr 2;16(4):e0249480. doi: 10.1371/journal.pone.0249480 (PMC8018634; doi:10.1371/journal.pone.0249480)
Supplement: S1 Appendix — (DOCX) [file pone.0249480.s001.docx]

**S1 Appendix: Multi-collinearity test results**

| Variable | VIF | 1/VIF |
| --- | --- | --- |
| Wealth | 3.05 | 0.327559 |
| Parity | 2.03 | 0.491450 |
| Age | 1.93 | 0.517379 |
| Partner’s education | 1.93 | 0.517495 |
| Education | 1.93 | 0.518700 |
| Residence | 1.84 | 0.542564 |
| Region | 1.54 | 0.650740 |
| Access to Mass Media | 1.51 | 0.663374 |
| Religion | 1.21 | 0.829575 |
| Marital status | 1.13 | 0.887651 |
| Getting medical help for self: distance to health facility | 1.11 | 0.903829 |
| Occupation | 1.06 | 0.945150 |
| Hold a valid NHIS card | 1.04 | 0.958850 |
| Ethnicity | 1.03 | 0.970952 |
| Health decision making capacity | 1.02 | 0.977342 |
| Mean VIF | 1.56 | |
