# Supplementary material for: Factors influencing early postnatal care utilisation among women: Evidence from the 2014 Ghana Demographic and Health Survey
Source: PLoS One. 2021 Apr 2;16(4):e0249480. doi: 10.1371/journal.pone.0249480 (PMC8018634; doi:10.1371/journal.pone.0249480)
Supplement: S2 Appendix — (DOCX) [file pone.0249480.s002.docx]

**S2 Appendix: Linktest results**

| LIN | Coef. | Std Err | Z | p>/z/ | 95% CI |
| --- | --- | --- | --- | --- | --- |
| _hat | 1.03 | 0.10 | 9.89 | 0.000 | [0.83-1.23] |
| _hatsq | 0.02 | 0.06 | 0.36 | 0.718 | [-0.09-0.13] |
| _cons | -0.01 | 0.07 | -0.09 | 0.927 | [-0.15-0.13] |
